# Network pharmacology-based analysis of the mechanism of *Saposhnikovia divaricata* for the treatment of type I allergy

**DOI:** 10.1080/13880209.2022.2086583

**Published:** 2022-06-27

**Authors:** Xiangsheng Li, Hui Li, Tingting Wang, Yang Zhao, Yuxin Shao, Yizhao Sun, Yanfen Zhang, Zhongcheng Liu

**Affiliations:** a College of Pharmaceutical Sciences, Key Laboratory of Pharmaceutical Quality Control of Hebei Province, Institute of Life Science and Green Development, Hebei University, Baoding, China; b Department of Urology, Peking University International Hospital, Beijing, China; c Technology Transfer Center, Hebei University, Baoding, China

**Keywords:** Traditional Chinese medicine, allergic targets, cell degranulation, IgE

## Abstract

**Context:**

*Saposhnikovia divaricata* (Turcz.) Schischk (Apiaceae) (SD) has various pharmacological activities, but its effects on type I allergy (TIA) have not been comprehensively studied.

**Objective:**

This study evaluates the treatment and molecular mechanisms of SD against TIA.

**Materials and methods:**

The effective components and action targets of SD were screened using TCMSP database, and allergy-related targets of SD were predicted using GeneCards and OMIM database. The obtained target intersections were imported into David database for GO analysis, and used R software to perform KEGG analysis. The RBL-2H3 cells sensitised by DNP-IgE/DNP-BSA were treated with different concentrations of SD (root decoction, 0.5, 1, and 2 mg/mL), prim-*O*-glucosylcimifugin (POG, 10, 40, and 80 μg/mL) and the positive control drug–ketotifen fumarate (KF, 30 μM) for 12 h, then subjected to cell degranulation and qPCR analysis.

**Results:**

Eighteen active compounds of SD and 38 intersection targets were obtained: TIA-related signal pathways mainly include calcium signal pathway, PI3K–Akt signal pathway and MAPK signal pathway. Taking the β-Hex release rate of the model group as the base, the release rate of SD and POG in high dose groups were 43.79% and 57.01%, respectively, which were significantly lower than model group (*p* < 0.01), and significantly lower than KF group (63.83%, *p* < 0.01, *p* < 0.05). SD and POG could down-regulate the expression of related proteins in the Lyn/Syk, PI3K/AKT and MAPK signalling pathways.

**Discussion and conclusion:**

*Saposhnikovia divaricata* could inhibit IgE-induced degranulation of mast cells, providing a scientific basis for further research and clinical applications of SD in TIA treatment.

## Introduction

With the development of industrialisation, lifestyle modernisation and environmental pollution, allergic diseases are increasing, affecting more than 20% of the global population (Abrams et al. 2021). However, the detailed mechanism is not yet fully elucidated. Allergic reactions can be divided into four types, each of which has a different mechanism. Among them, type I allergy (TIA, IgE-mediated) is the most clinically common (Liu et al. 2016), which is a complex immune response. The combination of IgE and FcεRI on the surface of mast cells (MC) or basophils is the key step, leading to aggregation and crosslinking reactions, which can induce MC or basophils releasing sensitising agents to cause allergy (Pedretti and Peter 2020; Shin et al. 2015).

At present, anti-TA drugs in the clinic mostly work by inhibiting the release of sensitising mediators or the expression of mediator genes (Meltzer et al. 2009), such as antihistamines, corticosteroids, anti-leukotrienes and so on. Although these drugs have immediate effects in various allergic diseases, they can’t fundamentally inhibit the occurrence of allergic reactions, and their clinical application is limited due to their defects and adverse reactions (Bahekar et al. 2008; Modgill et al. 2010; Urbagarova et al. 2020). The appearance of monoclonal anti-IgE antibody greatly increases the safety and effectiveness of the treatment of allergic diseases, but long-term use may lead to drug resistance of extreme medical expense (Kopp et al. 2009). Although people have learned more about allergy, the pathogenesis is not clearly understood, Therefore, it is necessary to continue to study the occurrence and development of allergy and find effective drugs with less adverse effects.

*Saposhnikovia divaricata* (SD), the dried roots of *Saposhnikovia divaricata* (Turcz.) Schischk (Apiaceae), called “Fang-feng” in China, is a traditional Chinese herbal medicine, listed as a top-grade Chinese medicine in Shen Nong's Herbal Classic (Chinese Pharmacopoeia Commission 2020). It grows mainly in the north and northeast of China, and is also planted in other provinces of China (Chun et al. 2016). SD is widely used in the clinic, and about 8% of prescriptions in the Chinese Pharmacopoeia contain SD. It is often used to treat headache, fever, arthritis and so on (Han et al. 2016). Researchers have isolated many effective components from SD, such as chromones, coumarins, acidic polysaccharides, volatile oils and so on. However, chromones and polysaccharides are considered as the main active substances of SD (Chun et al. 2016). Modern pharmacological studies have shown that SD has good anti-inflammatory and immune regulating effects, can effectively relieve rheumatoid arthritis and regulate the metabolic disorder of collagen-induced arthritis rats (Li et al. 2021). Prim-*O*-glucosylcimifugin (POG) is one of the main active substances of *Saposhnikovia* chromone, which can reduce the inflammatory reaction of RAW 264.7 macrophage induced by lipopolysaccharide (Zhou et al. 2017). SD is mostly used in compound preparations in the clinic. For example, Yupingfeng powder, a compound preparation of SD, can adjust intestinal flora balance of Rex rabbits, obviously improve immunity of Rex rabbits (Sun et al. 2016), and can also adjust Th1/Th2 imbalance and affect immune function of mice by regulating T cell differentiation (Wang et al. 2019). In addition, SD has antipyretic, analgesic, antitumor, and antioxidant effects (Meng et al. 2019; Wang et al. 2019; Ding et al. 2020; Urbagarova et al. 2020).

Many targets and pharmacological effects of SD are related to allergy, so we predict that it is an underlying drug for treating allergic diseases. At present, there is no detailed study on the treatment of TIA diseases by SD, and its therapeutic effect and mechanism still need to be explored in depth. Network pharmacology is a new drug research method based on multi-directional systematic pharmacological and biological analysis, which has become of great interest for pharmacological research (Trame et al. 2016). Network pharmacology of traditional Chinese medicine (TCM) is suitable for the characteristics of multi-target and complex pharmacological action of TCM, which has provided a good predictive platform for the mechanistic study of TCM.

In this paper, the underlying active components of SD and their interactive targets with allergic diseases were predicted by network pharmacology, and the possible signalling pathways were systematically analysed, and the results of network pharmacological analysis were verified by IgE induced cell degranulation simulated TIA. These results will provide clues for analysing the mechanism of SD in treating allergic diseases.

## Materials and methods

### Materials

The herbs of *Saposhnikovia divaricata* (whole root) was purchased in July 2020 from Beijing Solarbio Science & Technology Co., Ltd (Beijing, China), and collected from Heilongjiang Province, China. The rat basilic leukaemia cell line 2H3 (RBL-2H3) was obtained from Chinese Academy of Sciences. Ketotifen Fumarate (KF) was obtained from Sigma-Aldrich (St. Louis, MO, USA). DNP-IgE was obtained from Sigma-Aldrich. DNP-BSA was obtained from Biosearch. Primers were synthesised by Sangon Biotech Co., Ltd. (Shanghai, China). POG standard (purity (HPLC) ≥ 98.0%) was obtained from Beijing Solarbio Science & Technology Co., Ltd. (Beijing, China). Takara RNAiso Plus kit, reverse transcription agent cassette record and quantitative PCR kit were obtained from Takara.

Preparation of SD decoction: 2 g SD root standard was added 20 mL (10 times amount) of ultrapure water, soaked for 0.5 h, then refluxed in water bath at 100 °C for 1 h, and the filtrate was collected after suction filtration. 20 mL (10 times amount) of ultrapure water was added into filter residue, refluxed in water bath at 100 °C for 1 h, and the filtrates was collected together after suction filtration. A solution containing SD at a concentration of 50 mg/mL was obtained, and store at −20 °C after filtration with 0.22 μm microporous membrane.

### Network pharmacology analysis

#### Screening of potential active compounds

Chemical constituents of SD were searched using the TCM database and analysis platform (TCMSP, https://tcmspw.com/tcmsp.php). According to ADME related parameters, the potential active substances were screened (Oral bioavailability, OB ≥ 30%; Drug like index, DL ≥ 0.18). Finally, the collection of active ingredients of SD was obtained.

### Acquisition of targets

According to the screened active ingredients, TCMSP and SymMap (https://www.symmap.org/) database were used to search out the action targets of the active ingredients of SD, and the full name of the target gene was transformed into the symbol of the gene by Uniprot database (https://www.uniprot.org/), which was used for subsequent analysis. Entering the keyword Allergy in the databases of Gene Cards (https://www.genecards.org/) and Omim (https://www.omim.org/), and combining the target genes obtained from the two databases to get the set of allergy-related target genes (Zhang et al. 2010). Drawing Venn diagram (http://bioinformatics.psb.ugent.be/webtools/Venn/), and getting the intersection of action target and allergy target of active ingredients of SD.

### Construction of relationship network

The network of relationships among SD, active ingredients of SD, allergy and interaction targets was constructed by the software of Cytoscape 3.6.1 (https://cytoscape.org/). Firstly, the active ingredients of SD and the intersection targets of SD and allergy were sorted into text files. The input file of Cytoscape 3.6.1 software was generated by Perl (https://www.perl.org/) at the computer terminal, and imported into Cytoscape 3.6.1 software. Then, the data were visualised by Cytoscape 3.6.1 software to obtain the relationship network diagram between them.

### Construction of the protein–protein interaction (PPI) network

STRING protein***–***protein interaction online analysis database (https://string-db.org/) is a database integrating known and to-be-predicted protein***–***protein interactions, and has a powerful function of visualising information to display the interaction network system (Zhang et al. 2010). Importing the target of SD acting on allergy obtained in 2.2.2 into String database, selecting the species as *Homo sapiens* through the "Multiple proteins" function of String database, and clicking "SEARCH" to get the network diagram of PPI. Clicking "hide disconnected nodes in the network" to remove free points in PPI network, and exporting high-definition PPI network diagram in PNG format and TSV file, and processing the TSV file with R software (https://www.r-project.org/) to obtain core gene histogram of PPI network.

### The analysis GO enrichment analysis and KEGG pathway

Using David database (https://david.ncifcrf.gov/) to carry out GO (gene ontology) enrichment analysis on the action targets obtained, including three parts (Molecular function, MF; Biological process, BP; Cellular component, CC) were used for GO enrichment analysis, and Excel was used to visualise the results. The KEGG (Kyoto encyclopaedia of genes and genomes) pathway analysis of target genes was carried out by calling Bioconductor package through R software (https://www.r-project.org/), and the result was visualised. The signal pathway is screened according to *p* value, because the *p* value is too small for analysis, the *p* value is converted to –logP for analysis.

### Effect of SD on degranulation of RBL-2H3 cells

#### Cell culture

RBL-2H3 cells were cultured in DMEM (containing 10% foetal bovine serum, 100 U/mL penicillin and 100 µg/mL streptomycin).

### Cell degranulation examination

The release of β-hexosaminidase (β-HEX) was measured as a marker of degranulation. RBL-2H3 cells (3 × 10^5^ cells/well) were seeded into 24-well plates and randomly divided into a control group, a model group, a positive group (KF, 30 μM), a negative control, groups treated with SD (0.5, 1, and 2 mg/mL) and POG (10, 40, and 80 μg/mL). Twenty-four hours later, except for the negative group, each group was sensitised overnight by 500 μL DMEM containing 0.4 μg/mL anti-DNP-IgE antibody, while negative group was given 500 μL DMEM. Twelve hours later, the treated, positive and negative groups were replaced with 1 mL of DMEM (containing drugs), and the other groups were replaced with 1 mL of DMEM. After 12 h, the treated, positive and model groups were given 200 μL PIPES (containing 0.4 μg/mL DNP-BSA), and the other groups were given 200 μL PIPES. Incubate for 1 h and ice bath for 10 min, collect supernatant. A 50 μL aliquot of each sample was added to a 96-well plate, 50 μL β-HEX substrate solutions (*p*-nitrophenyl-*N*-acetyl-β-d-glucosaminide) was added, and the plate was incubated at 37 °C for 1 h. The reaction was stopped by adding 200 μL sodium carbonate buffer and the optical density (OD) value for the release of β-HEX was measured at 405 nm. The experiment was repeated six times. The inhibition rate of β-Hex in each group was calculated according to the following formula:

β−HEX release rate (%)=(treated groups−negative group)/(model group−control group)×100%

### Detection of gene expression during RBL-2H3 cells degranulation

In the same way as that described in above, the reaction was stopped on ice for 10 min, and total RNA was extracted by the TRIzol approach. A 1 μL sample of total RNA was checked for purity and concentration using NanoDrop 2000 spectrophotometer (Thermo Scientific, Waltham, MA, USA). RNA was reverse-transcribed into cDNA. The expression of signalling molecules was detected by quantitative real-time polymerase chain reaction (qPCR). The following primers were used:

Lyn-F: 5′-GCAGAGGGAATGGCATACATC-3′,

Lyn-R: 5′-GCAAGGCCAAAATCTGCAA-3′;

Syk-F: 5′-ACAATGGACTGAACCCTG-3′,

Syk-R: 5′-AGTAGCCTGAGCCAAGAA-3′;

PLCγ-F: 5′-GGACTATGGTGGGAAGAAGC-3′,

PLCγ-R: 5′-TTTGCCCTCAGGACGAAT-3′;

PI3K-F: 5′-ATACTTGATGTGGCTGACG-3′,

PI3K-R: 5′-CAATAGGTTCTCGGCTTT-3′;

Akt-F: 5′-TGGCACCGTCCTTGATACCC-3′,

Akt-R: 5′-CACGACCGCCTCTGCTTTGT-3′;

P38-F: 5′-GGACCTAAAGCCCAGCAA-3′,

P38-R: 5′-CAGCCCACGGACCAAATA-3′;

ERK-F: 5′-TCTCCCGCACAAAAATAAG-3′,

ERK-R: 5′-GGAAGGGGACAAACTGAAT-3′;

JNK-F: 5′-ACAATGGACTGAACCCTG-3′,

JNK-R: 5′-AGTAGCCTGAGCCAAGAA-3′;

GAPDH-F: 5′-CCCACTAACATCAAATGGGG-3′,

GAPDH-R: 5′-CCTTCCACAATGCCAAAGTT-3′.

### Statistical analysis

All data were presented as the mean ± standard deviation (SD). All statistical analyses were performed using SPSS 19.0 software. Differences among multiple groups were analysed using one- way analysis of variance. Statistical significance was defined as a *p* value of <0.05.

## Results

### Screening of repeated targets between active components of SD and allergy

**Figure 1.**
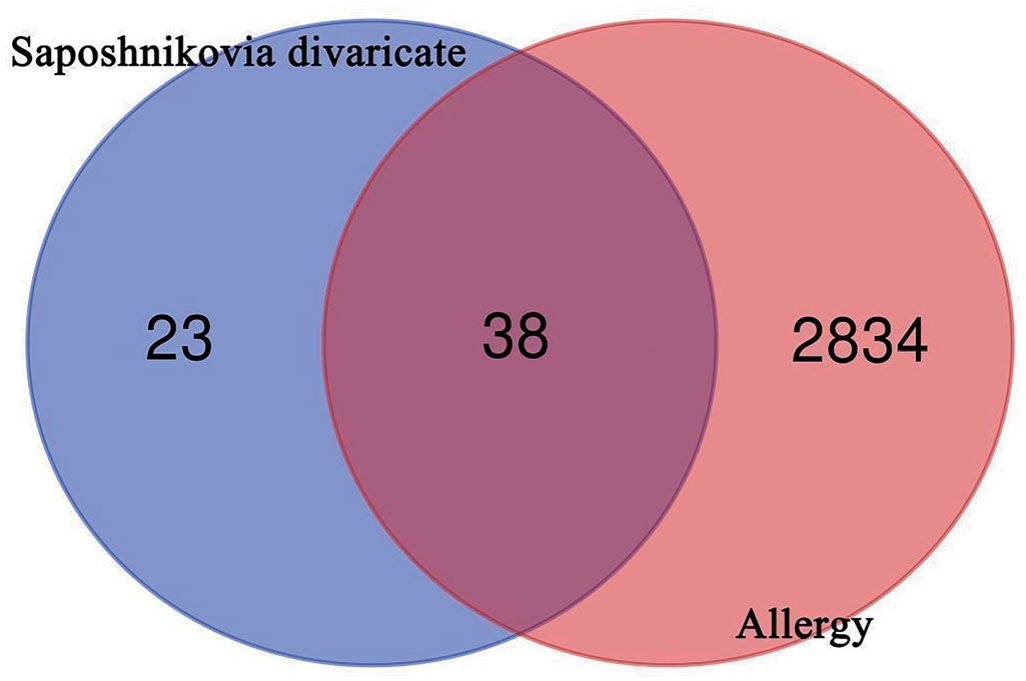
Venn diagram of the target of *Saposhnikovia divaricata* and allergy.

18 active ingredients of SD were collected from TCMSP database (OB ≥ 30%, DL ≥ 0.18) (Table 1), and 61 targets of SD and 2872 targets related to Allergy were collected. Then, the targets were merged and the repeated genes were removed. A total of 38 putative targets were identified, as shown in Table 2, Venn diagram was drawn (Figure 1).

**Table 1.** The main active ingredients of *Saposhnikovia divaricata*.

Mol ID	Molecule name	Molecular weight	OB (%)	DL
MOL000011	(2*R*,3*R*)-3-(4-hydroxy-3-methoxy-phenyl)-5-methoxy-2-methylol-2,3- dihydropyrano[5,6-h][1,4]benzodioxin-9-one	386.38	68.83	0.66
MOL011730	11-hydroxy-*sec-O-beta-*d-glucosylhamaudol_qt	292.31	50.24	0.27
MOL011732	anomalin	426.50	59.65	0.66
MOL011737	divaricatacid	320.32	87	0.32
MOL011740	divaricatol	334.35	31.65	0.38
MOL001941	ammidin	270.30	34.55	0.22
MOL011747	ledebouriellol	374.42	32.05	0.51
MOL011749	phelloptorin	300.33	43.39	0.28
MOL011753	5-*O*-Methylvisamminol	290.34	37.99	0.25
MOL002644	phellopterin	300.33	40.19	0.28
MOL000359	sitosterol	414.79	36.91	0.75
MOL000173	wogonin	284.28	30.68	0.23
MOL000358	beta-sitosterol	414.79	36.91	0.75
MOL001494	mandenol	308.56	42	0.19
MOL001942	isoimperatorin	270.30	45.46	0.23
MOL003588	prangenidin	270.30	36.31	0.22
MOL007514	methyl icosa-11,14-dienoate	322.59	39.67	0.23
MOL013077	decursin	328.39	39.27	0.38

**Table 2.** The potential targets of *Saposhnikovia divaricata* in allergy.

	Target name	Gene symbol
1	Nitric oxide synthase, inducible	NOS2
2	Potassium voltage-gated channel subfamily H member 2	KCNH2
3	Estrogen receptor	ESR1
4	Androgen receptor	AR
5	Prostaglandin G/H synthase 2	PTGS2
6	Vascular endothelial growth factor receptor 2	KDR
7	Prostaglandin G/H synthase 1	PTGS1
8	Peroxisome proliferator activated receptor gamma	PPARG
9	Acetylcholinesterase	ACHE
10	Estrogen receptor beta	ESR2
11	Muscarinic acetylcholine receptor M1	CHRM1
12	Retinoic acid receptor RXR-alpha	RXRA
13	Alpha-1A adrenergic receptor	ADRA1A
14	Alpha-1B adrenergic receptor	ADRA1B
15	Beta-2 adrenergic receptor	ADRB2
16	Mu-type opioid receptor	OPRM1
17	Progesterone receptor	PGR
18	Mitogen-activated protein kinase 14	MAPK14
19	Transcription factor p65	RELA
20	RAC-alpha serine/threonine-protein kinase	AKT1
21	Apoptosis regulator Bcl-2	BCL2
22	Caspase-9	CASP9
23	Transcription factor AP-1	JUN
24	Interleukin-6	IL6
25	Caspase-3	CASP3
26	Cellular tumour antigen p53	TP63
27	Interstitial collagenase	MMP1
28	C–C motif chemokine 2	CCL2
29	Prostaglandin E2 receptor EP3 subtype	PTGER3
30	Fibronectin	FN1
31	Interleukin-8	CXCL8
32	Muscarinic acetylcholine receptor M3	CHRM3
33	Muscarinic acetylcholine receptor M4	CHRM4
34	Muscarinic acetylcholine receptor M2	CHRM2
35	Sodium-dependent serotonin transporter	SLC6A4
36	Caspase-8	CASP8
37	Protein kinase C alpha type	PRKCA
38	Serum paraoxonase/arylesterase 1	PON1

### Construction of interaction network

**Figure 2.**
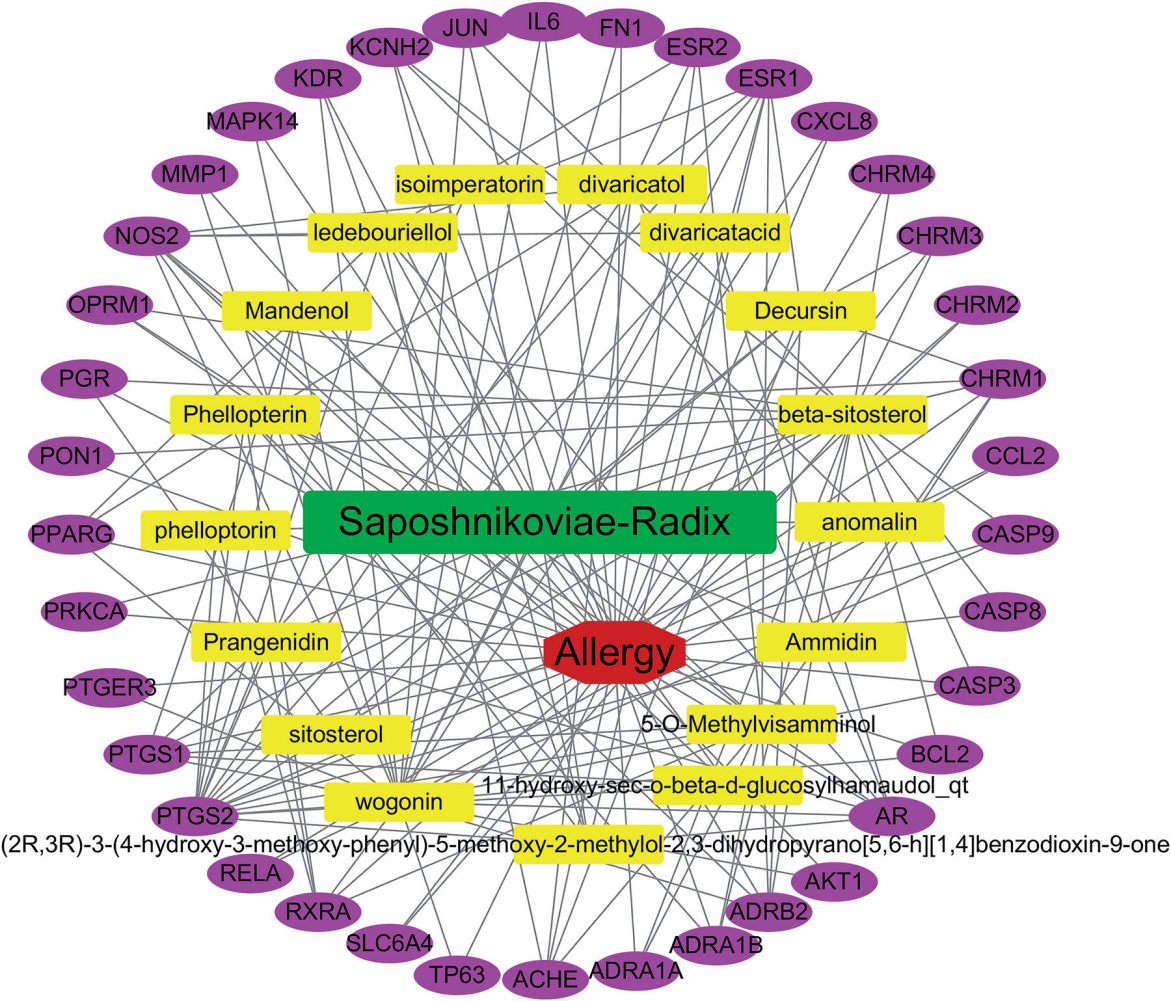
Network of *Saposhnikovia divaricata*–allergy–target. Red represents allergy, green represents *Saposhnikovia divaricata*, yellow represents active ingredients of *Saposhnikovia divaricata*, and purple represents intersecting target genes.

Using Perl and Cytoscape 3.6.1 software, we constructed the network diagram of SD, effective components of SD, allergy and the interactional targets (Figure 2). There are 57 nodes and 163 edges in the network, the more gene connection nodes, the more important they are. PTGS2 is the target with the highest correlation in the network, and most effective components of SD can act on this target. Wogonin is the component with the most associated targets in the network, which is associated with 22 targets. In the network diagram, the number of connections of a node is called degree, which reflects the importance of a node in the whole interaction network. The network analysis shows that the average degree of each compound is 5.7, which indicates that a single effective component of SD can act on multiple targets. The average degree of each target is 10.25, which indicates that each target may be associated with multiple components. It indicates the complexity of the TCM mechanism of action.

### Construction of PPI network

**Figure 3.**
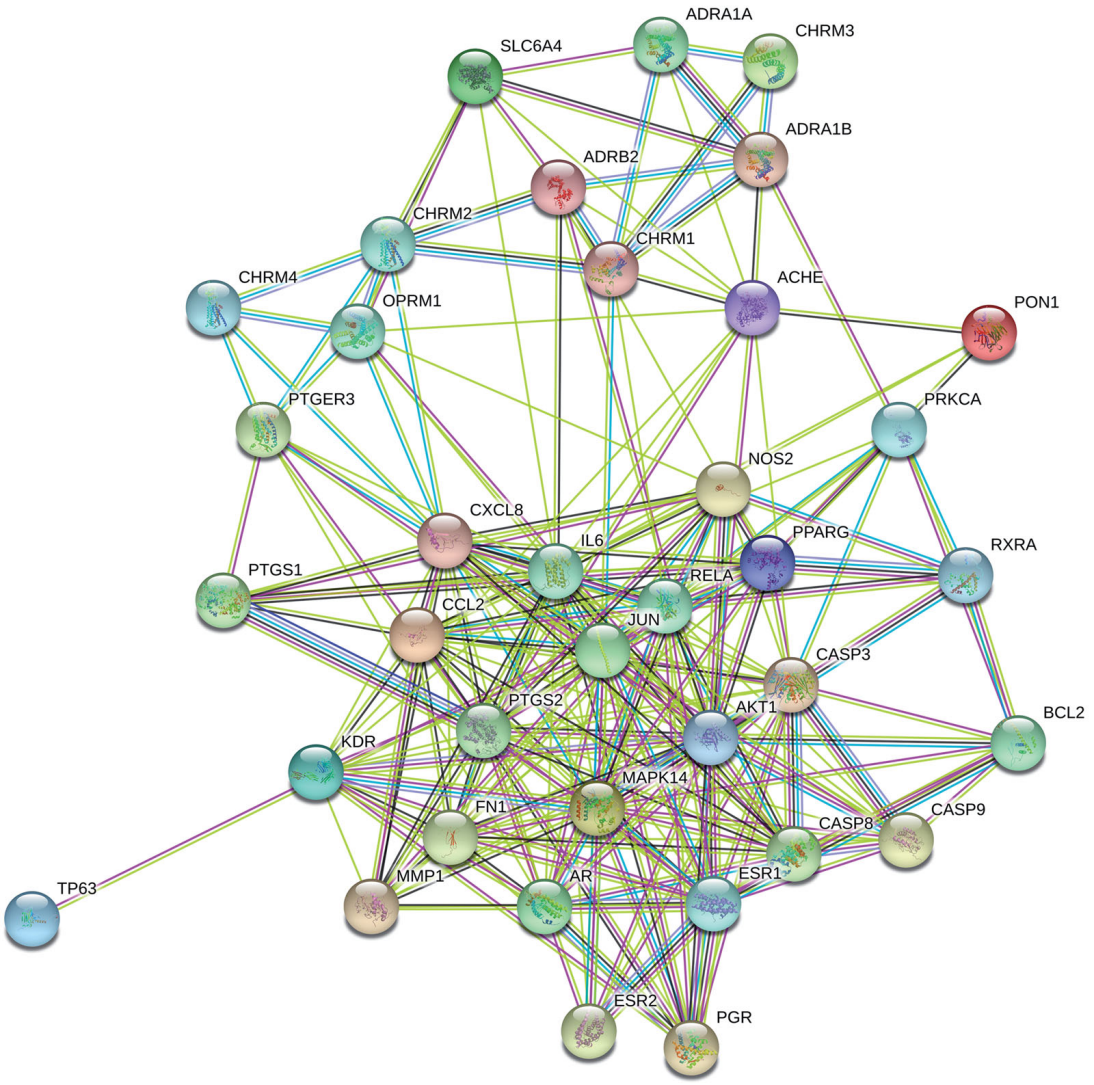
The PPI network of *Saposhnikovia divaricata*–allergy target. Light blue indicates known protein interactions in the database, purple indicates protein interactions found in experiments, green indicates adjacent genes, red indicates fusion genes, and blue indicates cooperative genes.

**Figure 4.**
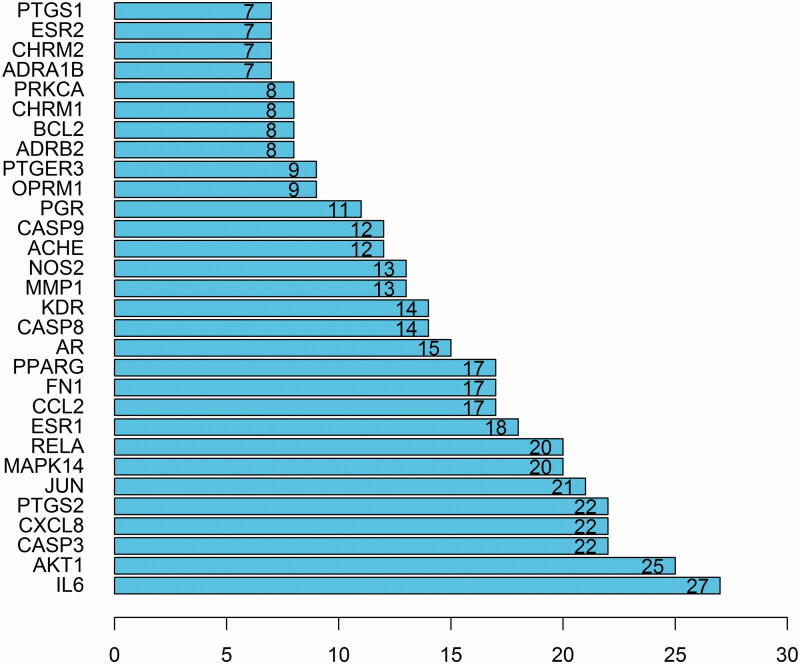
Core genes of PPI network.

The 38 target genes were imported into STRING database to construct PPI network (Figure 3). R software was used to screen out the top 30 genes with the most connection nodes in PPI network, and constructs bar graph (Figure 4). The genes with more connection nodes are IL6, AKT1, CASP3, CXCL8, PTGS2, JUN, MAPK14, RELA.

### GO enrichment analysis results

**Figure 5.**
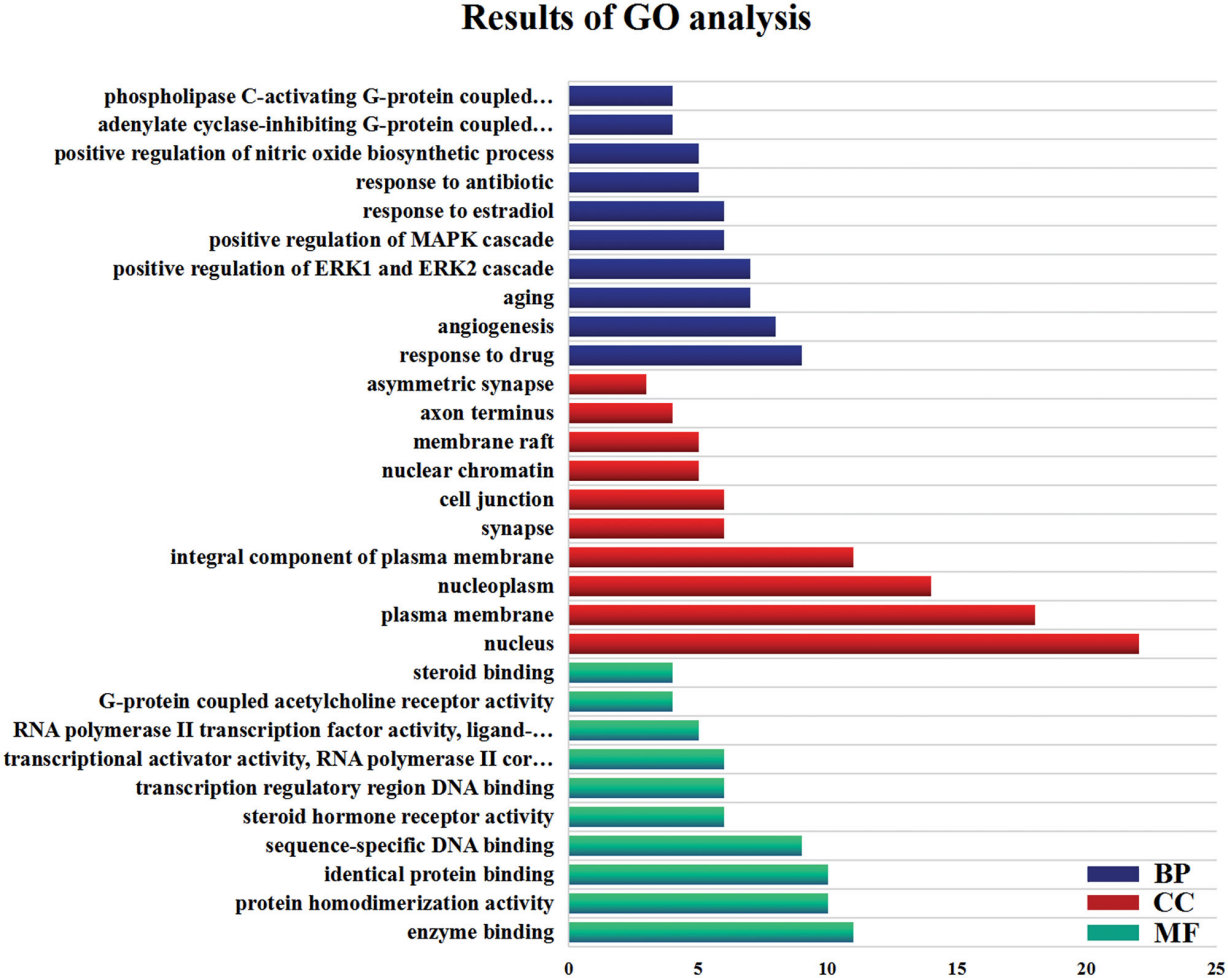
Results of GO analysis.

The 38 targets were introduced into David database for GO enrichment analysis (FDR ≤ 0.5), so as to systematically study the biological functions and related molecular processes that SD in treating allergy (Sivakumar et al. 2021). The results are shown in Table 3 that we obtained 194 GO–BP analysis results, 28 results of GO–CC analysis and 42 results of GO–MF analysis. The top ten results that involve the most targets were visualised in Figure 5.

**Table 3.** GO analysis of anti-allergic reactions of *Saposhnikovia divaricata*.

	Name	–LogP	Count
BP	Response to drug	6.589339	9
BP	Angiogenesis	6.312949	8
BP	Aging	5.82842	7
BP	Positive regulation of ERK1 and ERK2 cascade	5.67969	7
BP	Positive regulation of MAPK cascade	6.050565	6
BP	Response to oestradiol	5.798584	6
BP	Response to antibiotic	6.163517	5
BP	Positive regulation of nitric oxide biosynthetic process	5.6355	5
BP	Adenylate cyclase-inhibiting G-protein coupled acetylcholine receptor signalling pathway	6.46523	4
BP	Phospholipase C-activating G-protein coupled acetylcholine receptor signalling pathway	6.26177	4
CC	Nucleus	3.261846	22
CC	Plasma membrane	2.843911	18
CC	Nucleoplasm	2.635696	14
CC	Integral component of plasma membrane	3.414861	11
CC	Synapse	4.511398	6
CC	Cell junction	2.652976	6
CC	Nuclear chromatin	3.21278	5
CC	Membrane raft	3.106837	5
CC	Axon terminus	3.902681	4
CC	Asymmetric synapse	3.952939	3
MF	Enzyme binding	8.771268	11
MF	Protein homodimerization activity	4.676886	10
MF	Identical protein binding	4.588582	10
MF	Sequence-specific DNA binding	4.882256	9
MF	Steroid hormone receptor activity	6.871157	6
MF	Transcription regulatory region DNA binding	4.0189	6
MF	Transcriptional activator activity, RNA polymerase II core promoter proximal region sequence-specific binding	3.81003	6
MF	RNA polymerase II transcription factor activity, ligand-activated sequence-specific DNA binding	5.960986	5
MF	G-protein coupled acetylcholine receptor activity	6.472103	4
MF	Steroid binding	4.563169	4

### KEGG pathway analysis results

**Figure 6.**
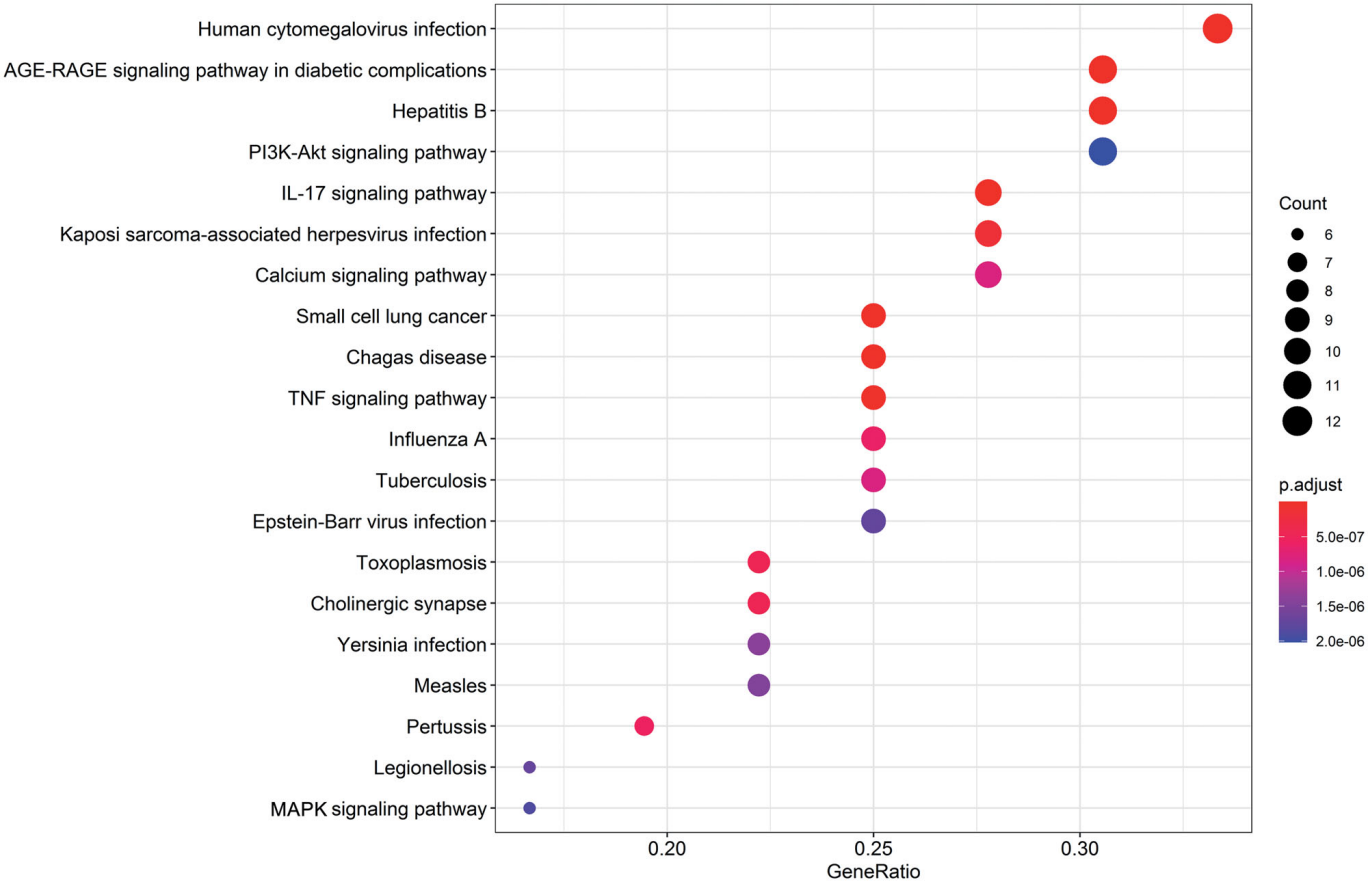
Enrichment analysis of pathways. The more target points involved in the pathway, the redder the colour, and the less target points involved, the bluer the colour. The ordinate indicates the name of the pathway involved.

Using R software to analyse the 38 targets by KEGG pathway enrichment (*P* value <0.05) (Liu et al. 2021), we obtained the 116 signalling pathways, then selected the top 20 signalling channels with significance (Table 4), and constructed bubble chart (Figure 6). The results showed that the signalling pathways related to TIA mainly include Calcium signalling pathway, PI3K–Akt signalling pathway, MAPK signalling pathway.

**Table 4.** KEGG analysis of anti-allergic reactions of *Saposhnikovia divaricata*.

NO.	Pathway name	–LogP	Gene number
1	Human cytomegalovirus infection	9.92132	12
2	AGE–RAGE signalling pathway in diabetic complications	12.5324	11
3	Hepatitis B	10.2087	11
4	PI3K–Akt signalling pathway	6.62582	11
5	IL-17 signalling pathway	11.2348	10
6	Kaposi sarcoma-associated herpesvirus infection	8.21448	10
7	Calcium signalling pathway	6.77456	10
8	Small cell lung cancer	9.78062	9
9	Chagas disease	9.37287	9
10	TNF signalling pathway	9.00612	9
11	Influenza A	7.38036	9
12	Tuberculosis	7.18716	9
13	Epstein–Barr virus infection	6.7561	9
14	Toxoplasmosis	7.61604	8
15	Cholinergic synapse	7.58551	8
16	Yersinia infection	7.10702	8
17	Measles	6.8804	8
18	Pertussis	7.46551	7
19	Legionellosis	6.7939	6
20	MAPK signalling pathway	5.56316	6

### Effects of SD on degranulation of RBL-2H3 cells induced by IgE

**Figure 7.**
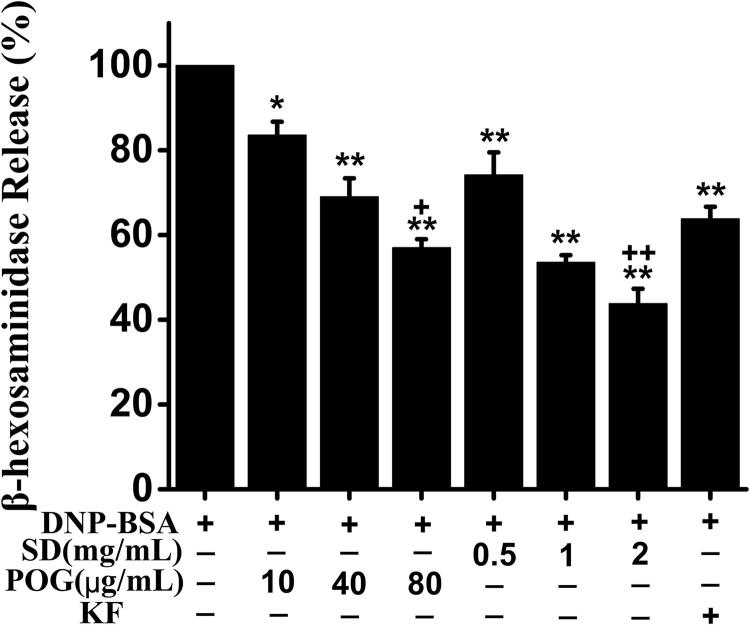
Effect of *Saposhnikovia divaricata* on the release of β-Hex from RBL-2H3 cells induced by DNP-IgE/DNP-BSA. ^##^*p* < 0.01 versus control; **p < 0.01 vs model; *p < 0.05 vs model; ^+^*p* < 0.05 vs KF; ^++^*p* < 0.01 vs KF.

β-Hex is a landmark substance for degranulation of MC. The results were shown in Figure 7. The release rate of β-Hex in the model group was the highest after the cells were stimulated, which indicated that the degree of cell degranulation was higher. After drug treatment, the release rate of β-Hex decreased significantly. Taking the β-Hex release rate of the model group as the base, the release rates of SD in low, middle and high dose groups were 74.18%, 53.58% and 43.79%, which were significantly lower than model group (*p* < 0.01). The release rates of POG in low, middle and high dose groups were 83.60%, 69.04% and 57.01%, which were significantly lower than model group (*p* < 0.05 or *p* < 0.01). The release rate of KF was 63.83%, which was also significantly lower than model group (*p* < 0.01). While POG high dose group and SD high dose group were significantly lower than KF group (*p* < 0.05, *p* < 0.01). The inhibitory effects of SD and POG on β-Hex release were concentration-dependent.

### Effects of SD on signalling molecules during degranulation of RBL-2H3 cells induced by IgE

The results of total RNA detection showed that the extracted RNA was complete and not degraded. According to the results of network pharmacological analysis, RT-qPCR was used to study the effects of SD on the expression of Lyn, Syk, PLCγ, PI3K, Akt, ERK, p38, JNK and other signalling molecules in the signalling pathway of TIA induced by DNP-IgE/DNP-BSA.

### Effects of SD on the expression of syk and lyn in IgE signalling pathway

**Figure 8.**
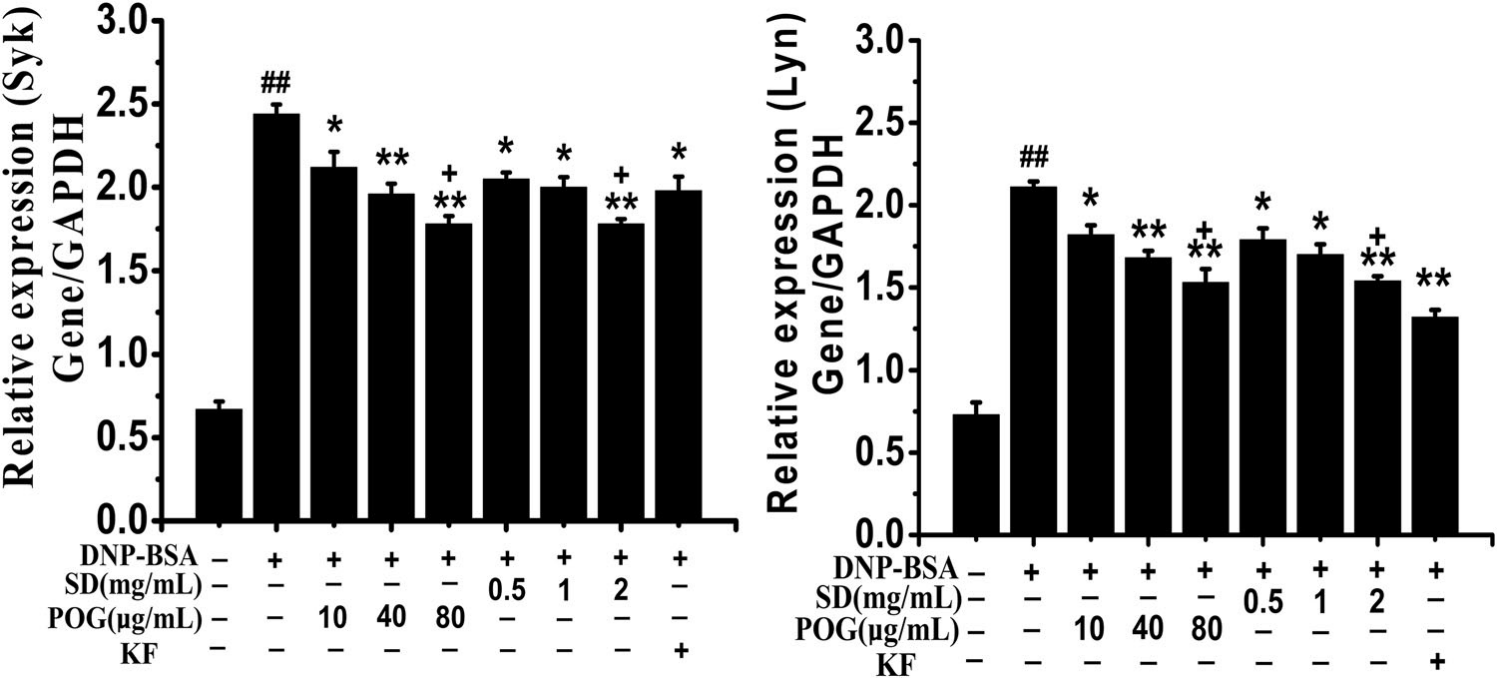
Effect of *Saposhnikovia divaricata* on the expression of Lyn, Syk, PLCγ in the DNP-IgE/DNP-BSA signalling pathway. ^##^*p* < 0.01 vs control; *p < 0.05, **p < 0.01 vs model; ^+^*p* < 0.05 vs KF.

Results as shown in Figure 8, the relative expression of Syk (Syk/GAPDH) in the model group was significantly higher than the control group (*p* < 0.01), and the relative expression of Syk decreased in different degrees after SD and POG treatment. The POG group was lower than the model group (*p* < 0.05, *p* < 0.01). The SD groups were lower than the model group (*p* < 0.05, *p* < 0.01). The inhibitory effect of the high-dose groups of SD and POG on Syk was higher than the KF positive group (*p* < 0.05). The inhibitory effect of SD and POG on Lyn expression was similar to Syk, but the inhibitory effect of SD and POG on Lyn was lower than the KF positive group (*p* < 0.05).

### Effects of SD on the expression of PI3K, AKT and PLCγ in IgE signalling pathway

**Figure 9.**
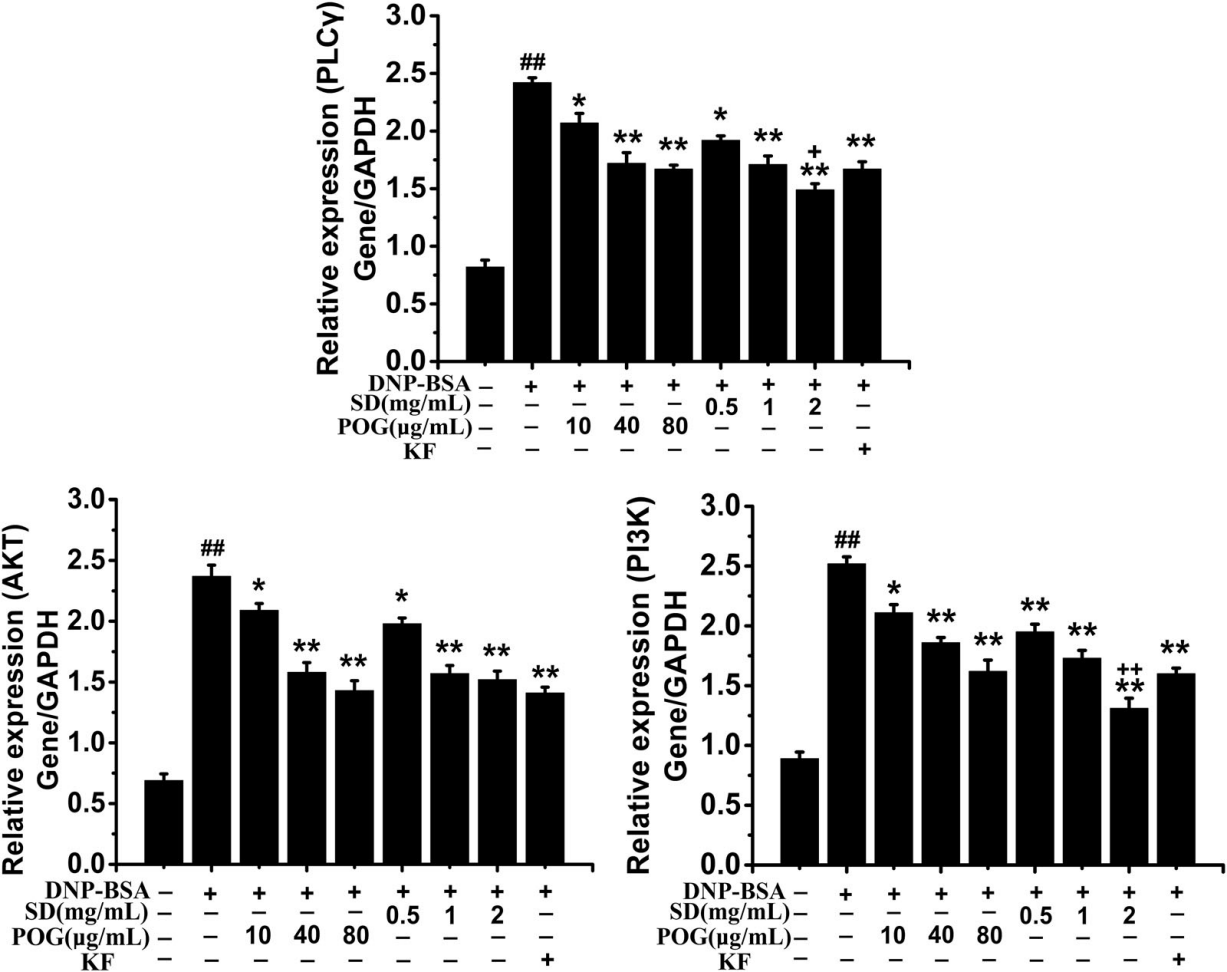
Effect of *Saposhnikovia divaricata* on the expression of PI3K and AKT in the DNP-IgE/DNP-BSA signalling pathway. ^##^*p* < 0.01 vs control; *p < 0.05, **p < 0.01 vs model; ^++^*p* < 0.01 vs KF.

Results as shown in Figure 9, the relative expression of PI3K (PI3K/GAPDH) in the model group was significantly higher than the control group (*p* < 0.01), while both SD groups and POG groups were significantly lower than the model group (*p* < 0.05, *p* < 0.01). The SD high-dose group was significantly lower than the KF positive group (*p* < 0.01), but there was no significant difference between POG high-dose group and KF positive group. The effect of POG and SD on Akt was similar to PI3K, while the inhibitory effect of KF positive group on Akt expression had no significant difference with the high-dose groups of POG and SD. The effect of drugs on PLCγ was similar to Akt, but the relative expression of PLCγ in KF positive group was higher than that in SD high-dose group (*p* < 0.05), but close to the POG high-dose group.

### Effect of SD on MAPK signalling pathway induced by IgE

**Figure 10.**
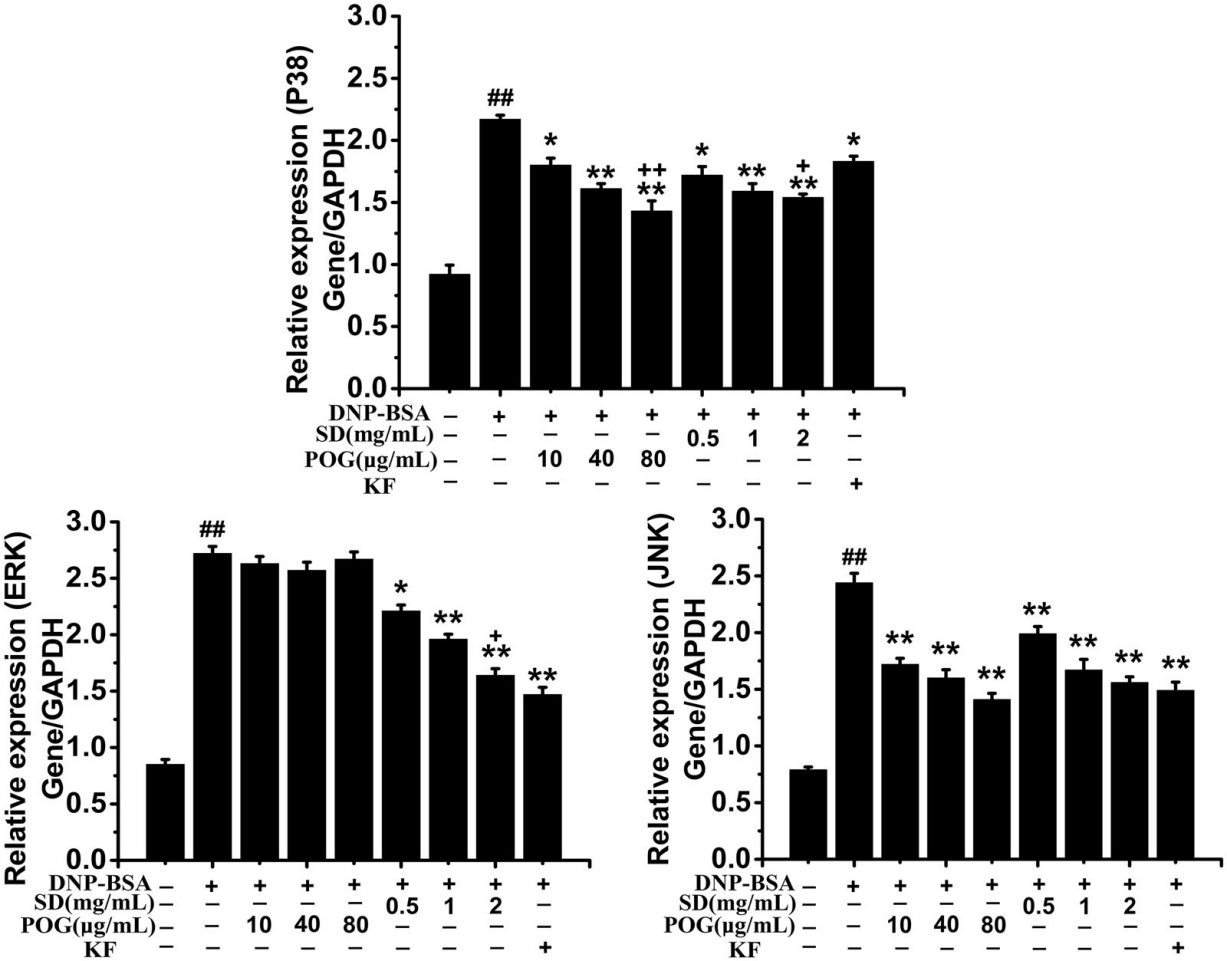
Effects of *Saposhnikovia divaricata* on MAPK signal pathway in RBL-2H3 cells induced by DNP-IgE/DNP-BSA. ^##^*p* < 0.01 vs control; *p < 0.05, **p < 0.01 vs model, ^+^*p* < 0.05, ^++^*p* < 0.01 vs KF.

Results as shown in Figure 10, POG and SD can inhibit the expression of P38 (P38/GAPDH), the inhibitory effect of KF positive group was lower than the SD high-dose group (*p* < 0.05), and significantly lower than POG high-dose group (*p* < 0.01). The relative expression of ERK in the model group was significantly higher than the control group (*p* < 0.01). After POG treatment, the relative expression of ERK did not decrease obviously. There is no significant difference between the POG dose groups and model group. After SD and KF treatment, the relative expression of ERK decreased significantly. The SD low-dose group was lower than the model group (*p* < 0.05), while the SD middle and high dose groups were significantly lower than the model group (*p* < 0.01). The inhibitory effect of SD high-dose group on ERK expression was lower than the KF positive group (*p* < 0.05). The relative JNK expression in all drug groups was significantly lower than in the model group (*p* < 0.01). However, there was no difference between KF treatment group and the high-dose groups of SD and POG.

## Discussion

SD is a complex TCM, and its main components and molecular mechanism have yet to be explored. Especially, there was little research on the mechanism of treating allergic diseases, so it was necessary to further explore its treatment mechanism. In the study on SD in the treatment of allergic reaction, researchers mostly judge the therapeutic effect of SD on allergic reaction by apparent phenomena. In recent years, it has been reported that SD may regulate the immune function of mice by regulating Th1/Th2 imbalance (Yu et al. 2015). However, the current studies have failed to explore the molecular mechanism of TCM in allergy. Network pharmacology and TCM network pharmacology provide a good prediction platform for studying the mechanism of action of TCM, has become the focus of TCM pharmacology (Trame et al. 2016). In this paper, the possible mechanism of SD in treating TIA diseases was predicted by network pharmacology, in order to obtain some new and valuable results.

Selecting the appropriate database is the basis of the success of network pharmacological analysis, while TCMSP database and TCMID (Traditional Chinese Medicine Integrated Database) database are commonly used to search Chinese medicine (Xue et al. 2013). In contrast, TCMSP database is the most commonly used retrieval tool for network pharmacology analysis of TCM. The database contains perfect drug data, including Chinese herbal medicines, herbal components, drug targets and related diseases. It records the structure, physical and chemical properties and pharmacokinetic data of TCM components in detail, which is the first choice for network pharmacology study of TCM (Dai et al. 2020; Zhang et al. 2021).

In addition, TCMSP database can provide a variety of pharmacokinetic parameters. OB value and DL value are the most commonly used reference data for drug screening. Generally, when the drug OB value is greater than or equal to 30%, it indicates that the oral bioavailability is good and it is easy for the body to absorb and play a role (Kim et al. 2021). The value of DL reflects the possibility of the compound becoming a drug. When DL is greater than or equal to 0.18, it is showed that the compound has the higher drug-like property and possibility of becoming a drug (Kim et al. 2021). In this paper, 18 underlying effective components of SD were screened using TCMSP database according to OB ≥ 30% and DL ≥ 0.18. At present, it has been reported that anomalin can inhibit the growth of tumour cells and release inflammatory mediators by inhibiting NF-κB, TNF-α and MAPK signalling pathways (Khan et al. 2013). In addition, ammidin can inhibit the activation of Th2 cells and may have the underlying to treat Th2-mediated allergic asthma (Lin et al. 2016). Wogonin is another potential active substance of SD, can significantly inhibit the inflammatory reaction induced by cadmium stimulation and show certain antioxidant and anti-apoptosis effects (Hong et al. 2020). Other studies have shown that Ecursin has anti-inflammatory and immune-regulating effects (Han et al. 2018). In short, many underlying active ingredients of SD have showed certain anti-inflammatory and immune-regulating effects, which are related to the mechanism of allergy. Therefore, SD has an underlying therapeutic effect on allergy, which takes effect through various pharmacological mechanisms.

Chromones, coumarins, acidic polysaccharides and volatile oils are the main effective components isolated from SD (Chun et al. 2016). It is generally believed that the main active ingredients of SD are POG, 5-*O*-methylvisamminol, cimicifugin, etc. POG and 5-*O*-methylvisammino are taken as the quality evaluation indexes of SD in Pharmacopoeia of the People's Republic of China (2020 edition) (Chun et al. 2016; Commission 2020). However, in the process of network pharmacological analysis, we found a worthy problem for attention. It showed that POG was not included in the list of ingredients of SD in TCMSP database. The lack of POG in TCMSP database indicates that the current network pharmacological analysis database of TCM needs to be continuously updated and improved. In current reports, POG is one of the index components of SD, and SD is the only source of POG. Chromones and polysaccharides are considered as the main active substances of SD, while POG is the chromone with the highest content in SD (Kong et al. 2013; Matusiewicz et al. 2019). According to reports, POG could significantly inhibit the production of nitrite and the expression of iNOS and COX-2 in RAW 264.7 cells induced by lipopolysaccharide, and showed significant anti-inflammatory effect (Zhou et al. 2017). However, when we searched the medicinal components of SD, POG was not classified as the effective component of SD in the TCMSP database, nor was this compound included, and the oral bioavailability and Drug like index of POG were not searched in other databases. Therefore, we also studied the anti-allergic effect of POG *in vitro*.

Thirty-eight overlapping targets between SD and allergic were obtained, and the network diagram showed that Wogonin had the most components connected with the target points. It was mentioned above that Wogonin had many pharmacological effects such as anti-inflammatory, anti-oxidation and anti-apoptosis (Hong et al. 2020), which indicated that Wogonin may be the key active ingredient of SD. The PPI network screened out the core targets closely related to the treatment of allergic reactions by windproof, and among those core targets, IL6, AKT1, CASP3, CXCL8, PTGS2, JUN, MAPK14 and RELA were ranking relatively high and at the core of the network. In the study of allergy mechanism, it was found that inflammation plays a role in the whole process of allergy occurrence and development, and interleukin plays an important role in promoting it (Dong et al. 2018; Xie and Dent 2019). IL-6 is a multifunctional cytokine, can promote inflammatory reaction through IL-6/JAK2/STAT3 signalling pathway (Azouz et al. 2020). CXCL8 can bind to IL8RA and IL8RB, which are chemokine receptors of CXCL8. After binding, it can make the neutrophils chemotactic movements, and thus regulate inflammatory response (Mund et al. 2018). It is reported that MAPK14 plays a key role in activating the expression of pro-inflammatory genes in vascular smooth muscle cells, which is dependent on p65/NF-κB pathway (Wu et al. 2019). In addition, RELA gene is the coding gene of NF-κB/P65 subunit protein, and is very important for the formation of NF-κB that has a wide range of functions in the body, such as inflammation, differentiation, immunity and apoptosis (Oliver Metzig et al. 2020; Kolesnichenko et al. 2021). AKT1, CASP3, PTGS2, JUN can participate in apoptosis, inflammation, and tumour occurrence (Mohamadzade et al. 2021; Liang et al. 2021; Pietruszewska et al. 2021). These target genes are related to allergic reactions to some extent, which also suggests that SD may have anti-apoptosis and antitumor effects in addition to its potential therapeutic effects on allergic reactions.

David database is a biological information database with a great deal of data, which can clearly show the enrichment of target genes through the analysis and integration of biological information about target genes, and is a common tool for bioinformatics analysis (Zhou et al. 2019). GO enrichment analysis and KEGG pathway analysis are commonly analytical methods in network pharmacology, and are the key steps to reveal the mechanism of action between drugs and diseases (Wang et al. 2020). GO analysis can link the gene catalogue obtained from the completely sequenced genome with the system functions at higher levels of cells, species and ecosystems, while KEGG analysis can screen the target proteins, find the signalling pathways involved by drug targets, and explain the potential mechanism of drug treatment of diseases. Through two analysis methods, the molecular function, biological process and influence on disease-related signalling pathway of drug action can be clarified. IgE/FcεRI signalling pathway is the key signalling transduction pathway to trigger TIA (Wang et al. 2020). The results of KEGG enrichment analysis showed that SD may play a role in the treatment of allergic reaction by influencing interleukin-17, Ca^2+^ signal, PI3K/Akt and MAPK. There were all related to IgE/FcεRI signalling pathway. Therefore, the results indicated that SD may play a role in the treatment of TIA diseases through multi-component and multi-target to regulate of Ca^2+^ signal, PI3K/Akt signalling pathway and MAPK signalling pathway, because these signal pathways have the potential to be closely related to allergy.

The occurrence of TIA is affected by many factors. MC is one of its main effector cells, which have become the main research object in the field of allergy research. However, the number of mast cells *in vivo* is small, the stability *in vitro* is poor, and the extraction process is complicated, so there is no mature extraction method at present. RBL-2H3 cells are the most commonly used MC substitute model in scientific research. They have similar bioactive factor release pathways and biological functions. After activation, RBL-2H3 cells could release a variety of bioactive factors, including histamine, leukotriene and β-Hex etc. Many reports showed that β-Hex could be released stably during degranulation, and its released concentration is relatively high, which was beneficial to biological detection, so it had become a common detection index for degranulation of RBL-2H3 cells (Chen et al. 2018; Han et al. 2018).

Here, we induced RBL-2H3 cell degranulation model by DNP-IgE/DNP-BSA, and prepared SD water decoction by decocting, which was the most common pharmaceutical method in TCM. At the same time, we explored the anti-allergic effect of cimicifugin as an index component of SD (Dong et al. 2018; Commission 2020). We studied the inhibitory effects of the two drugs on cell degranulation, and detected the related signalling molecules according to the results of network pharmacology, so as to clarify the mechanism of SD in treating TIA. As the positive control, Ketotifen Fumarate is a commonly anti-allergic drug in clinic, which has the function of stabilising MC and remarkable anti-allergic effect. At present, it has become a common positive control drug in allergy research (Chen et al. 2021).

It was found that SD could effectively inhibit the release of β-Hex from RBL-2H3 cells induced by IgE. Lyn and Syk are the key signalling molecules upstream of IgE. When TIA occurs, Lyn can phosphorylate its adjacent protein tyrosine after being activated, thus further improve the recruitment degree and activation of Lyn and Syk (Bugajev et al. 2010). Activated Syk can phosphorylate many cytoplasmic signalling molecules, such as PLCγ, PI3K, Akt, etc. Activation of PLCγ can act on PIP2 to generate signalling molecule IP3. The binding of IP3 to its receptor can lead to the increase of intracellular Ca^2+^ concentration, which eventually leads to the degranulation reaction of MC (Kraft and Kinet 2007). Therefore, this study suggested that SD could inhibit Ca^2+^ release by regulating Lyn/Syk pathway, thus inhibiting MC degranulation.

Both SD and POG inhibited the expression of Akt and PI3K in cells to some extent, but the inhibitory intensity of POG was weaker than SD. The reason could be that POG was a monomer while SD was a prepared decoction having complex components, other components in SD may also play a certain role, which components play the major role needs further analysis. When TIA occurs, activated PI3K produces phosphoinositide-3,4,5-triphosphate, which activates PLCγ through aggregation binding protein tyrosine kinase (such as Akt), resulting in Ca^2+^ inflow, cell degranulation and release of inflammatory mediators (Biethahn et al. 2014). It has been found that PI3K/Akt signalling pathway plays a key role in allergic reaction. In addition, Wu found that the ethanol extract of *Cordyceps sinensis* can inhibit the phosphorylation of PI3K, thereby inhibit mast cell degranulation and reduce the release of allergic mediators (Wu et al. 2017). This study showed that SD could also relieve the symptoms of TIA by inhibiting PI3K/Akt pathway.

In addition, MAPK is another key signalling pathway that regulates the release of allergenic mediators and cytokines in allergic reactions. MAPK signalling transduction modules are divided into at least three modules: ERK 1/2, p38 MAPK and JNK 1/2, which jointly regulate the physiological functions of cells (Xu et al. 2021). Therefore, this study detected the expression of key genes in intracellular MAPK signalling pathway using qPCR. The results showed that SD and POG inhibited the expression of p38 and JNK to some extent. However, only SD had a certain inhibitory effect on ERK expression, while POG had no obvious inhibitory effect on ERK expression. At the same time, some studies have pointed out that isoimperatorin could also block the phosphorylation of ERK-MAPK/AP1 signalling pathway to prevent MC degranulation to yield an anti-inflammatory function (Ahmad et al. 2020). Isoimperatorin is an important monomer component of SD. When SD and POG acted on cells, POG may be unable to inhibit ERK expression by itself, while SD could inhibit ERK expression because of containing isoimperatorin. Generally speaking, SD may play a role in treating allergy by inhibiting MAPK signalling transduction, which is consistent with the results of network pharmacological analysis.

## Conclusions

The anti-allergic mechanism of SD was predicted by network pharmacology, and verified the molecular mechanism of anti-TIA of SD with cell model. The results showed that both the SD decoction and POG may regulate TIA mainly through MAPK signalling pathway, and it may also involve Lyn/Syk and PI3K/AKT signalling pathways to some extent to inhibit IgE-induced degranulation of mast cells. The results also confirmed that POG was an important effective medicinal component of SD. Furthermore, SD indicated the multi-dimensional regulatory mechanism of anti-allergy, this study results has provided valuable clues for further study of the underlying mechanism.
